# Supplementary material for: Depletion of LONP2 unmasks differential requirements for peroxisomal function between cell types and in cholesterol metabolism
Source: Biol Direct. 2023 Sep 22;18:60. doi: 10.1186/s13062-023-00416-3 (PMC10515011; doi:10.1186/s13062-023-00416-3)
Supplement: Supplementary file 1 — Additional file 1. Supplementary figures. [file 13062_2023_416_MOESM1_ESM.docx]

# Supplementary Figure Legends

## Figure S1. LONP2 knockdown impaired protein homeostasis of the full-length ACOX1 in COS-7 cells.

Immunoblotting for ACOX1 antibody in *NT*- and *LONP2*-silenced COS-7 cells after 144 h. ACOX1-F and ACOX1-P indicate the full-length ACOX1 and the processed forms of ACOX1, respectively. Vinculin was used as a loading control.

## Figure S2. LONP2 knockdown triggered luminal protein import failure in U2OS cells.

Representative confocal images (left) and quantification (right) of the colocalization between peroxisomes (PMP70) and transiently expressed peroxisome luminal protein (CFP-SKL) in non-targeting (NT)- and *LONP2*-silenced U2OS cells after 144 h. Scale bar, 2 µm. Dots represent individual cells from *n* = 3 technically independent experiments depicted in different colours, and triangles represent their respective means. The mean values were used to calculate the average (horizontal bar), s.d. (error bars), and *P*-values (using a two-tailed unpaired Student’s *t*-test).

## Figure S3. LONP2 knockdown did not change the fate of catalase in COS-7 cells.

Immunoblotting of *NT*- and *LONP2*-silenced COS-7 cells for catalase (a) and representative confocal images of the colocalization between catalase and peroxisomes (PMP70) in *NT-* and *LONP2*-silenced COS-7 cells after 144 h (b). Vinculin was used as a loading control. Scale bars, 10 µm.

## Figure S4. Superoxide accumulation in mitochondria was detected in *LONP2*-silenced COS-7 cells.

Representative confocal images (left) and quantifications (right) of the colocalization between MitoSOX-stained mitochondrial superoxide (green) and TMRE-stained mitochondria (magenta). Scale bars, 10 µm. Data from *n* = 3 technically independent experiments (30 cells per experiment), and their respective means are depicted in triangles. The mean values were used to calculate the average (horizontal bar), s.d. (error bars) and *P*-values (using a two-tailed unpaired Student’s *t*-test).

## Figure S5. Peroxisomal gene expression is not significantly altered upon loss of LONP2.

Volcano plots showing expression of peroxisomal genes in both COS-7 (left) and U2OS (right). Selected peroxins linked to peroxisomal biogenesis are highlighted with red dots.

## Figure S6. Differences and commonalities of lipidomic changes between COS-7 cells and U2OS cells.

Volcano showing all 2,094 lipid features retained in the final dataset for the comparison between COS-7 and U2OS cells under steady-state conditions (**a**) and 7-day of *LONP2*-silenced vs. NT-silenced COS-7 cells (**b, left**) or U2OS cells (**b, right**). X-axes show fold-change (FC) (log_2_) in MS signal intensity values and y-axes the corresponding corrected p-values (-log_10_*P_corr_*) following testing using unpaired Student *t*-test followed by a Benjamini-Hochberg correction. Lipid features that significantly passed our selected thresholds as indicated by red dotted lines (horizontal: *P_corr_* value <0.05; and vertical │(FC)│> 1.5)) were annotated to unique lipids using MS/MS and data alignment with an in-house database (see Table S2 for details). Colors indicated lipid subclasses as indicated in the small inset. Grey dots indicate features that have not been identified. (**c)** Dot plots for sphingomyelin (SM(d34:0)) and cholesterol ester (CE(20:1) or CE(22:1)) in both COS-7 cells and U2OS cells. Data from *n* = 5 technically independent experiments. Abbreviations: free fatty acid (FFA), diacylglycerol (DG), triacylglycerol (TG), monoacylglycerophosphocholine (LPC), diacylglycerophosphocholine (PC), 1-alkyl, 2-acylglycerophosphocholine (PCO-), 1-(1Z-alkenyl), 2-acylglycerophosphocholine (PCP-), monoacylglycerophosphoethanolamine (LPE), diacylglycerophosphoethanolamine (PE), 1-(1Z-alkenyl), 2-acylglycerophosphoethanolamine (PEP-), diacylglycerophosphoinositol (PI), diacylglycerophosphoglycerol (PG), sphingomyelin (SM), ceramide (Cer), glucosylceramide (GlcCer), cholesterol derivative (chol der) and cholesterol ester (CE).

## Figure S7. Overloaded free cholesterol accumulated within lysosomes at different time courses in *LONP2*-silenced U2OS cells after 7 days.

Representative confocal images showing the Filipin-stained cholesterol (green) and endolysosomes (magenta) visualized with the immunofluorescence of LAMP1 at different time courses (control, 2h, 6h, 24h) after the addition of free cholesterol in *NT-* and *LONP2*-silenced U2OS cells. Scale bars, 10 µm.

## Figure S8. U2OS cells with excess free cholesterol did not show the activation of CHOP upon silencing of LONP2.

Immunoblotting of *NT*- and *LONP2*-silenced U2OS cells for LONP2 and CHOP at different time courses (control, 2h, 6h, 24h) after the addition of free cholesterol. Vinculin was used as a loading control.

## Figure S9. PEX5 silencing reveals common and distinct changes relative to siLONP2.

Immunoblotting of non-targeting (*NT*)- and *PEX5*-silenced COS-7 cells and U2OS cells, examining CHOP (ISR), CRABP2 (RA signaling), INSIG1 (cholesterol handling), and RRS1 (ribosomal biogenesis). GAPDH used as a loading control.

Figure S1


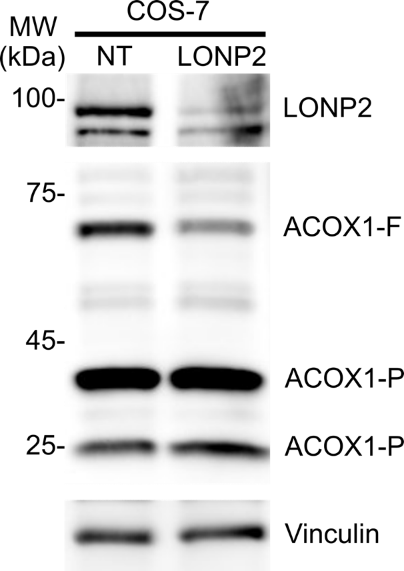


Figure S2


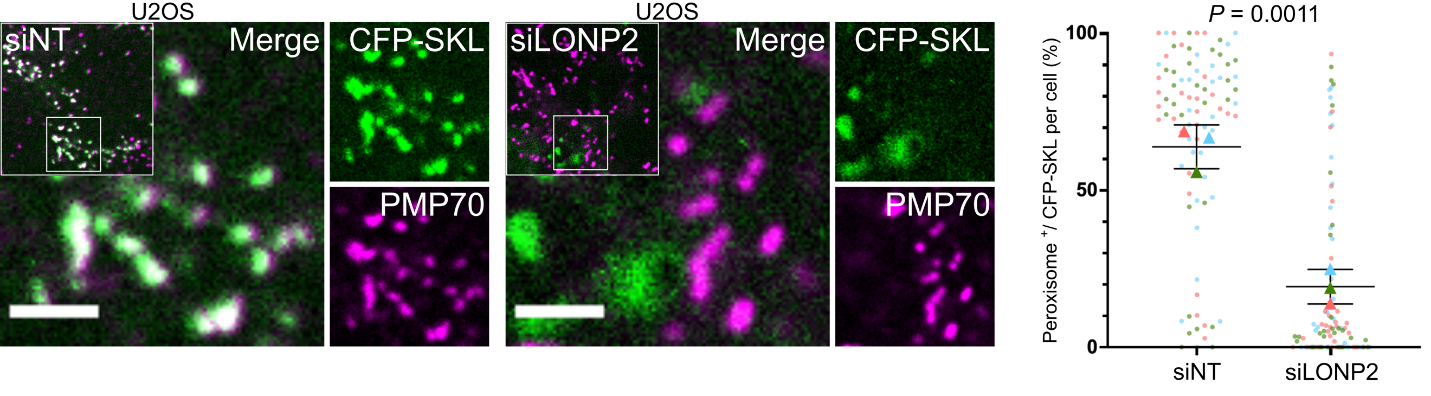


Figure S3


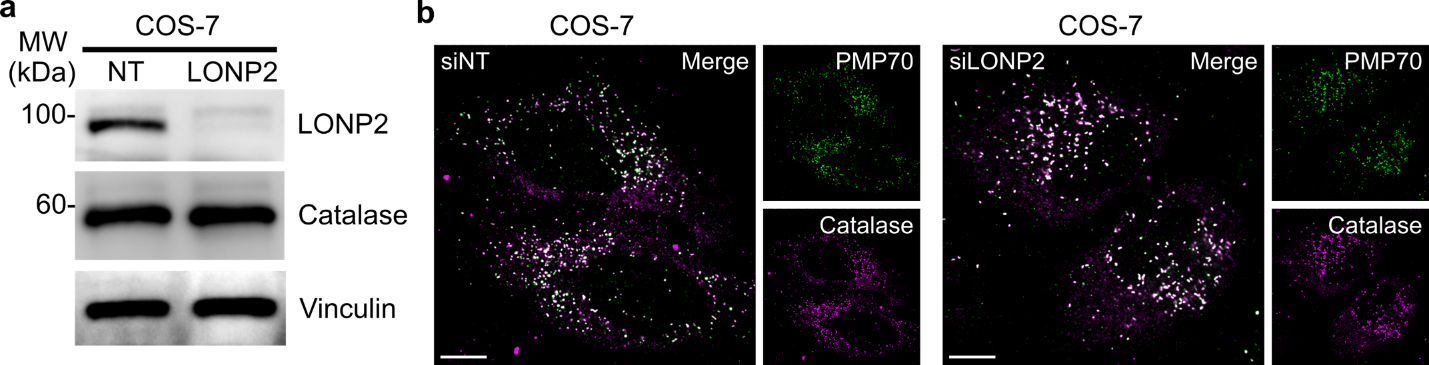


Figure S4


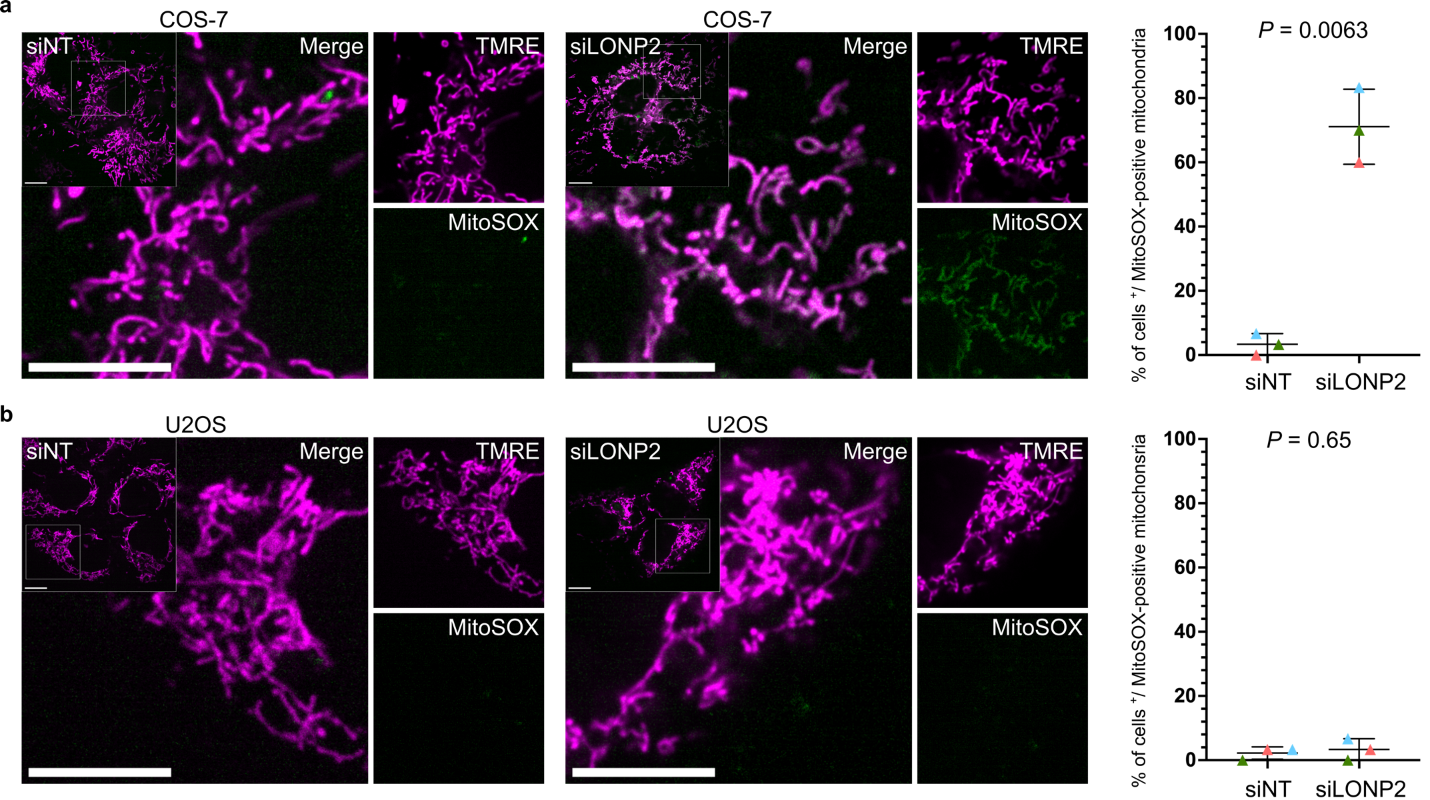


Figure S5


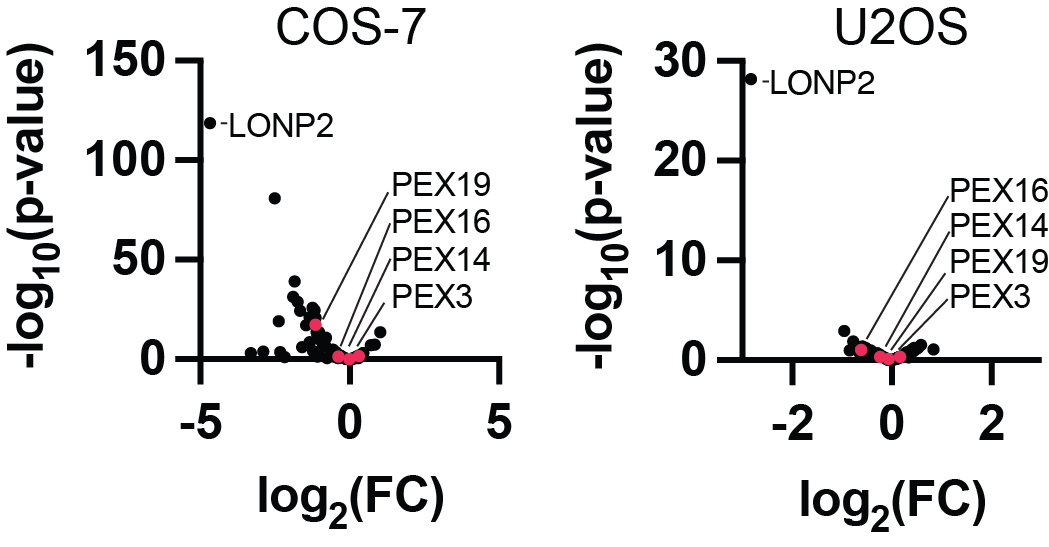


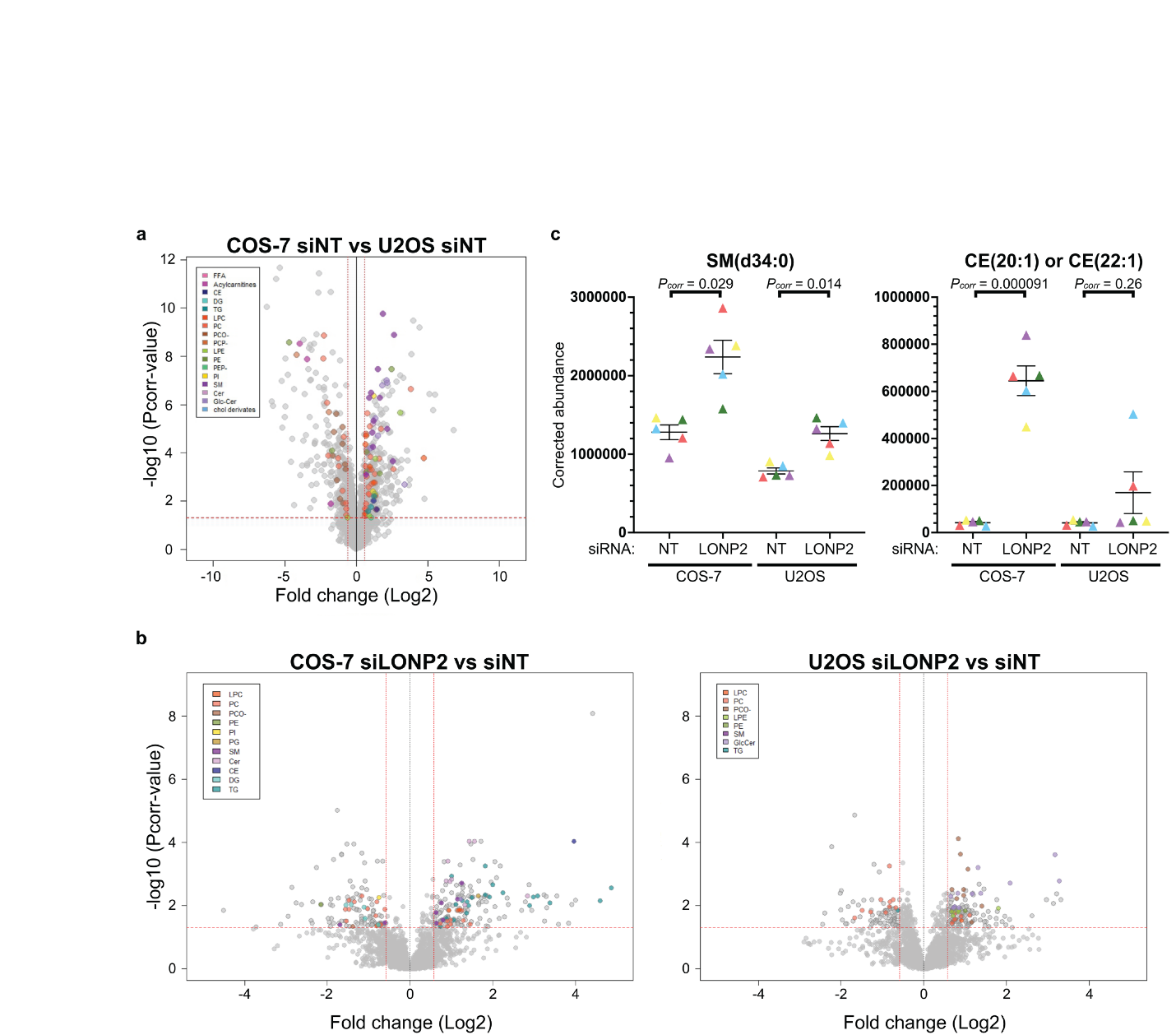
Figure S6

Figure S7


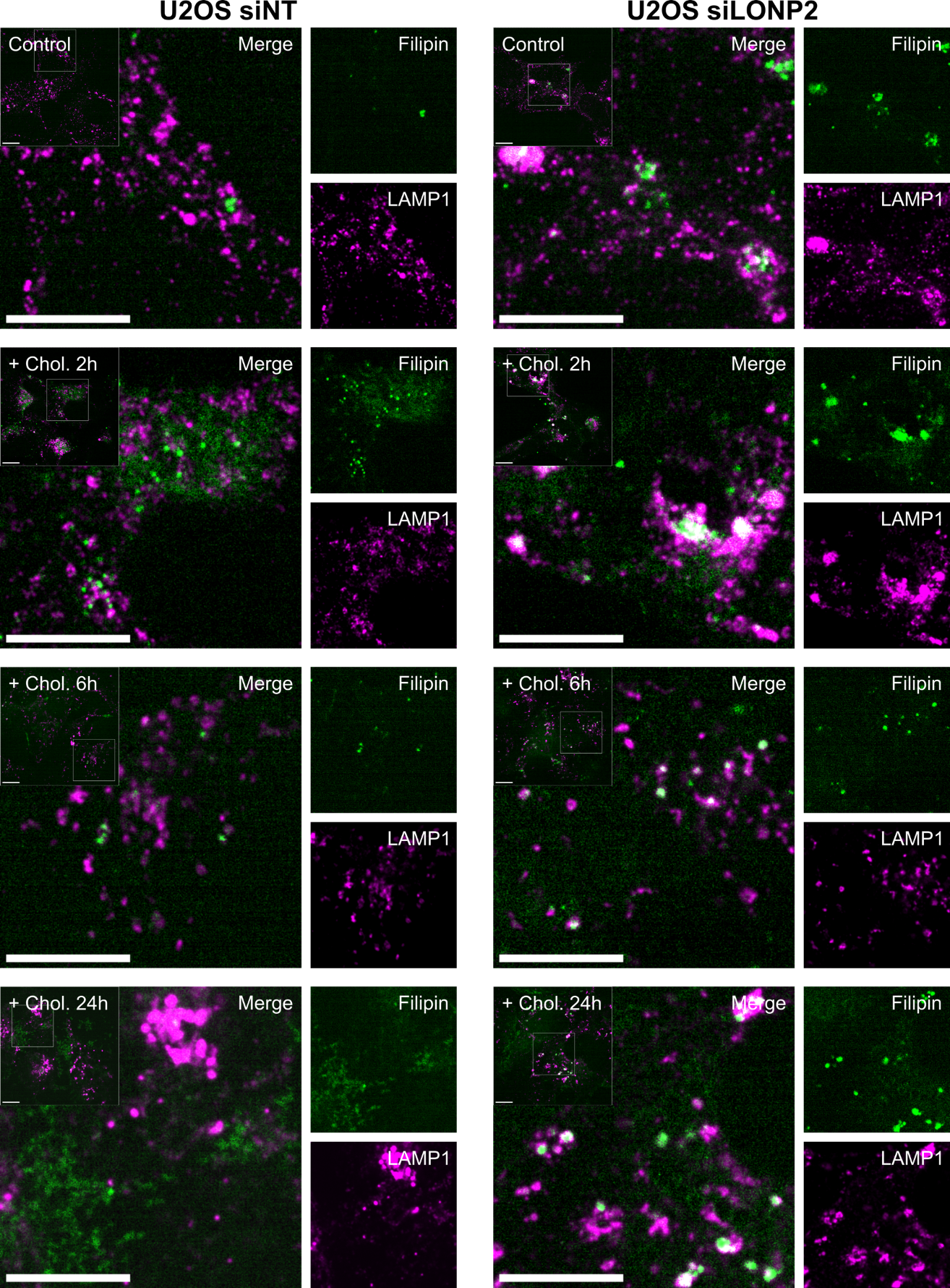


Figure S8


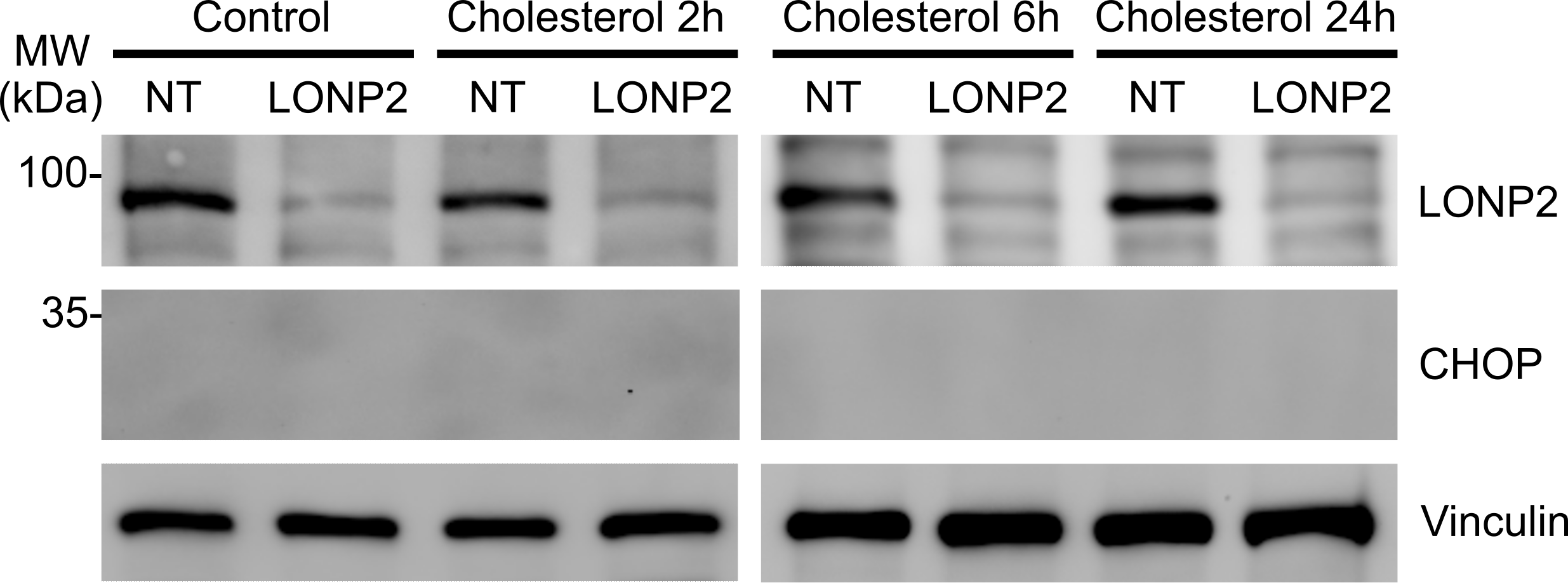


Figure S
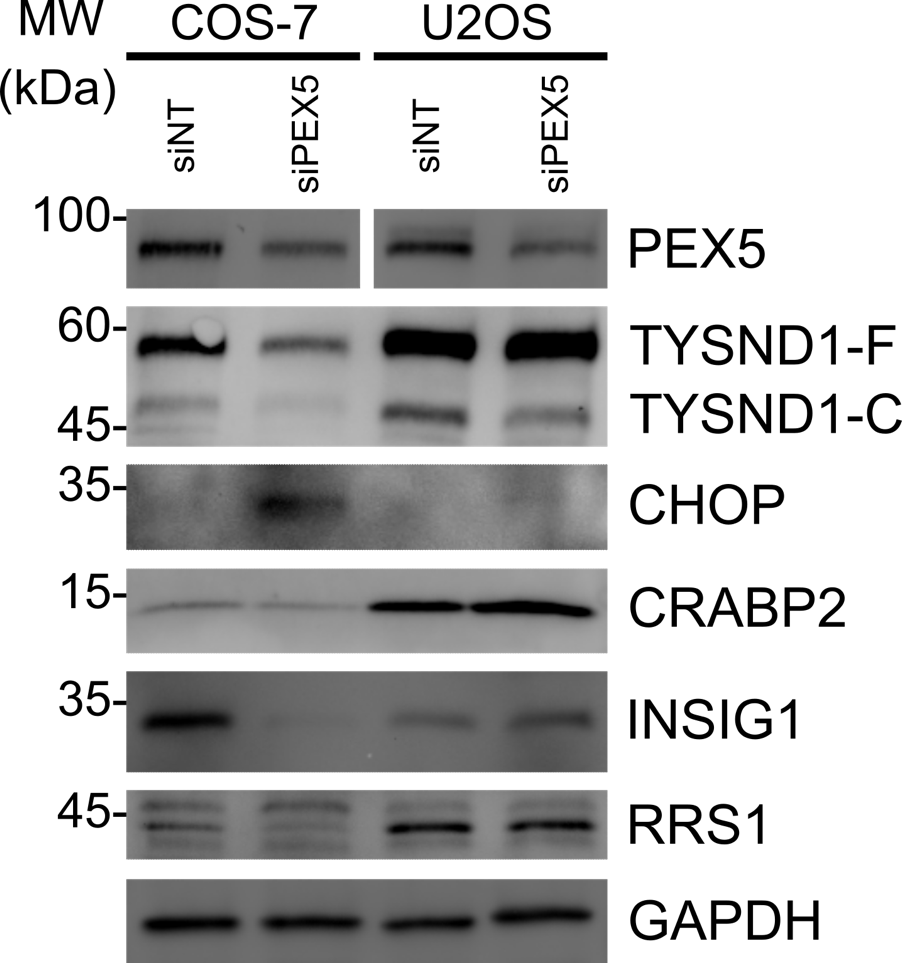
9
